# Modeling Spatio-Temporal Dynamics of Optimum Tilt Angles for Solar Collectors in Turkey

**DOI:** 10.3390/s8052913

**Published:** 2008-05-06

**Authors:** Can Ertekin, Fatih Evrendilek, Recep Kulcu

**Affiliations:** 1 Department of Agricultural Machinery, Akdeniz University, Faculty of Agriculture, 07070, Antalya, Turkey; 2 Department of Environmental Engineering, Abant Izzet Baysal University, Faculty of Engineering & Architecture, 14280, Bolu, Turkey; 3 Department of Agricultural Machinery, Suleyman Demirel University, Faculty of Agriculture, 32260, Cunur, Isparta, Turkey

**Keywords:** Solar radiation, Solar collector, Spatio-temporal modeling, Optimum tilt angle

## Abstract

Quantifying spatial and temporal variations in optimal tilt angle of a solar collector relative to a horizontal position assists in maximizing its performance for energy collection depending on changes in time and space. In this study, optimal tilt angles were quantified for solar collectors based on the monthly global and diffuse solar radiation on a horizontal surface across Turkey. The dataset of monthly average daily global solar radiation was obtained from 158 places, and monthly diffuse radiation data were estimated using an empirical model in the related literature. Our results showed that high tilt angles during the autumn (September to November) and winter (December to February) and low tilt angles during the summer (March to August) enabled the solar collector surface to absorb the maximum amount of solar radiation. Monthly optimum tilt angles were estimated devising a sinusoidal function of latitude and day of the year, and their validation resulted in a high *R*^2^ value of 98.8%, with root mean square error (RMSE) of 2.06°.

## 1. Introduction

Turkey receives a high level of solar radiation throughout the year with mean daily sunshine duration of about 7.2 h and solar energy intensity of 12.96 MJ m^-2^ d^-1^. The highest and lowest solar energy potential of Turkey is in the Southeast Anatolian region with an average solar radiation of 14.37 MJ m^-2^ d^-1^ and sunshine duration of 8.2 h d^-1^ and in the Black Sea region with an average solar radiation of 11.02 MJ m^-2^ d^-1^ and sunshine duration of 5.4 h d^-1^, respectively [1]. The solar potential unconstrained by technical, economic or environmental requirements of Turkey is estimated at 88 million tones oil equivalent (toe) per year, 40% of which is considered economically usable. Three-fourths (24.4 million toe per year) of the economically usable potential is considered suitable for thermal use, with the reminder (8.8 million toe per year) for electricity production [2].

Although Turkey has high potential and untapped renewable energy resources, Turkey is an energy importing country due to its heavy reliance on fossil fuels and limited availability of indigenous fossil energy resources. Turkey had primary energy production of 25.1 million toe and primary energy consumption of 91.5 million toe in 2005. The present trend of the imbalance between the production and consumption of energy continues to increase each year. Total solar energy production of 5000 toe in 1986 increased to 385,000 toe in 2005 and is projected to rise to 5.5 million toe (Mtoe) (5.5% of primary energy production) by 2025 [3, 4]. Flat plate solar collectors are the most widespread solar thermal application in Turkey, which are generally used for the production of commercial and domestic hot water, especially throughout the coastal regions. In 2005, Turkey had 11 million m^2^ of collector surface area installed with a heat output of 0.4 Mtoe contributing to energy production [5].

Given the target by the EU of 500 m^2^ solar collector for every 1000 citizens, and the present Turkish collector manufacturing capacity of 1 million m^2^ per year, the growth of solar thermal market is expected to continue, thus increasing the quantity and quality of collectors installed on Turkish roofs and greenhouses [6-8]. The performance of the solar collector is highly dependent on its orientation, optical and geometric properties, macro and microclimatic conditions, geographical position, and the period of use [9-13]. The orientation of the solar collector is described by its azimuth (γ) and tilt angle relative to the horizontal and considered to be optimal when facing south (γ = 0°) in the northern hemisphere. The optimum tilt angle depends on latitude (λ), solar declination or days of the year [12, 13]. Daily solar energy collected was reported to be 19 to 24% higher by a solar PV panel with one axis east-west tracking system than by a fixed system [14]. Since the solar tracking systems have high operation and maintenance costs and are not always applicable, it is often convenient to set the solar collector at an optimum tilt angle over time [15].

Various optimum tilt angles were determined for such systems in the literature as follows: λ + 15° for winter months (October to March) [16]; λ + 20° [17]; λ + (10-30)° [18]; λ + 10° [19, 20]; λ - 10° [21]; λ ± 20° [22]; λ ± 8° [23]; λ ± 5° [24]; λ = β_opt_[ 25-27]; λ ± 15° [15, 28] and (λ + 15°) ± 15° [29] (the signs “+”, “-”, and “β_opt_” denote for winter and summer months, and optimum tilt angle, respectively). Changing the optimum tilt angle for the latitudes between 0 and 60° by about ±10° and ±20° was reported to reduce the amount of the monthly absorbed radiation by about 2-3% and 6%, respectively [30, 31]. Qui and Riffat [32] suggested the tilt angle of the solar collector set within the optimum tilt angle of ±10° as an acceptable practice since the deviation from the maximum solar energy gain is below 1.5%.

The objective of this study was to devise a simple algorithm to quantify spatio-temporal dynamics of optimal tilt angles of the solar collectors in Turkey for the maximization of energy collection.

## 2. Data and Methodology

### 2.1. Observed Data

In this study, the geo-referenced dataset of monthly average daily global radiation on a horizontal surface from 158 weather stations in Turkey between 1968 and 2004 was used to estimate monthly, seasonally, and annually optimum tilt angles and explore the relationship among optimum tilt angle (degrees), day of the year, and latitude (decimal degrees).

### 2.2. Description of Algorithm

Monthly averages of the daily global solar radiation incident on a horizontal surface are available for many locations; however, global solar radiation data on tilted surface are lacking in many locations, and thus, need to be calculated. Total solar radiation on a tilted surface (*H*_t_) consists of direct or beam radiation (*H*_b_) (MJ m^-2^ d^-1^), diffuse radiation (*H*_d_) (MJ m^-2^ d^-1^) and ground reflected radiation (*H*_r_) (MJ m^-2^ d^-1^). Monthly collectable radiation on a tilted surface for a given month (MJ m^-2^ d^-1^) can be estimated as follows [29, 33]:

(1) $$H_{\text{t}}=H_{\text{b}}+H_{\text{d}}+H_{\text{r}}$$

(2) $$H_{\text{t}}=(H-H_{\text{d}})R_{\text{b}}+\frac{H_{\text{d}}}{2}(1+\cos\beta )+\frac{H}{2}\rho (1-\cos\beta )$$

The equations (1 and 2) can be simplified as follows:

(3) $$H_{\text{t}}=R.H=R.K_{T}.H_{\text{o}}$$

where *R* is defined as the ratio of daily average radiation on a tilted surface to that on a horizontal surface for each month and can be expressed as follows [33]:

(4) $$R=\left(1-\frac{H_{d}}{H}\right)R_{b}+\frac{H_{d}}{2H}(1+\cos\beta )+\frac{\rho }{2}(1+\cos\beta )$$

The monthly average clearness index (*K*_T_) is the ratio of monthly average daily radiation on a horizontal surface (*H*) (MJ m^-2^ d^-1^) to monthly average daily extraterrestrial radiation on a horizontal surface (*H*_o_) (MJ m^-2^ d^-1^). *H_o_* can be calculated from the following equation [34]:

(5) $$H_{o}=\left(\frac{24}{\pi }\right)I_{gs}f\left[\cos\lambda .\cos\delta .\sin w_{s}+\left(\frac{\pi }{180}\right).w_{s}.\sin\lambda .\sin\delta \right]$$

where *I*_gs_ is the solar constant (1367 W m^-2^); *f* the eccentricity correction factor; λ latitude; δ the solar eclination (degrees); and *w*_s_ the mean sunrise hour angle for a given month. The eccetricity correction factor, solar declination and sunrise hour angle can be computed thus [29]:

(6) $$f=1+0.033\left(\cos\frac{360.n}{365}\right)$$

(7) δ=23.45sin[360(284+n)/365]

(8) $$w_{s}=\cos^{-1}\left(-tg\lambda .tg\delta \right)$$

where *n* is the number of the day of the year starting from the first of January. In order to determine monthly average daily diffuse solar radiation over Turkey, the following correlation developed by Tasdemiroglu and Sever [35] was used:

(9) $$\frac{H_{d}}{H}=1.6932-8.22262\left(\frac{H}{H_{o}}\right)+25.5532{\left(\frac{H}{H_{o}}\right)}^{2}-37.807{\left(\frac{H}{H_{o}}\right)}^{3}+19.8178{\left(\frac{H}{H_{o}}\right)}^{4}$$

*R*_b_ is a function of the transmittance of the atmosphere and can be estimated as the ratio of extraterrestrial radiation on the tilted surface to that on a horizontal surface for a given month. For surfaces directly facing the equator [33]:

(10) $$R_{b}=\frac{\cos(\lambda -\beta ).\cos\delta .\sin w_{s}^{\prime }+\left(\frac{\pi }{180}\right).w_{s}^{\prime }.\sin(\lambda -\beta ).\sin\delta }{\cos\lambda .\cos\delta .\sin w_{s}+\left(\frac{\pi }{180}\right).w_{s}.\sin\lambda .\sin\delta }$$

where $W_{s}^{'}$ is the sunset hour angle for the tilted surfaces and estimated thus [33]:

(11) $$w_{s}^{\prime }=\min\{w_{s},\text{arccos}\left[-tg(\lambda -\beta ).tg\delta \right]\}$$

Spatial interpolation of annual optimum tilt angles was created for the entire Turkey of 780,580 km^2^ using the deterministic interpolation method of inverse distance weighting (IDW) through the ArcGIS geostatistical analyst module 9.1 [36]. Inverse distance weighting estimates values of unknown surfaces as a function of distance-weighted averages of values of measured points within a defined neighborhood surrounding the unmeasured points, with points closer to the prediction locations having more influence on the predicted values than points located farther away as follows [37]:

(12) $$\overset{\wedge }{z}(x_{o})=\sum_{i=1}^{N}\lambda_{i}.z(x_{i})$$

where

(13) $$\lambda_{i}=\frac{d_{i0}^{-p}}{\sum_{i=1}^{N}d_{i0}^{-p}}\;\text{and}\;\sum_{i=1}^{N}\lambda_{i}=1$$

where *ẑ* is the predicted value at the unsampled point *x*_0_; N the number of measured sample points within the neighborhood defined for *x*_0_; *λ_i_* the distance-dependent weights associated with each sample points; *z*(*x_i_*) the observed value at point *x_i_*; d*_i0_* the distance between the prediction location *x*_0_ and the measured location *x*_i_; and *p* the power parameter that defines the rate of reduction of the weights as distance increases.

## 3. Results and Discussion

### 3.1. Monthly Optimum Tilt Angles

**Figure 1.**
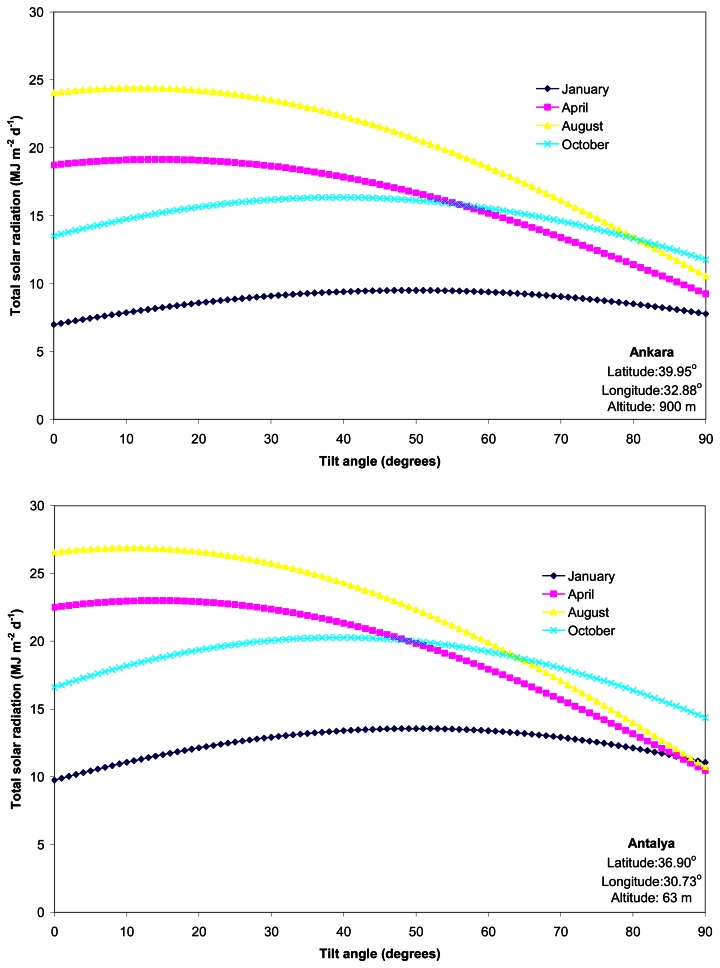

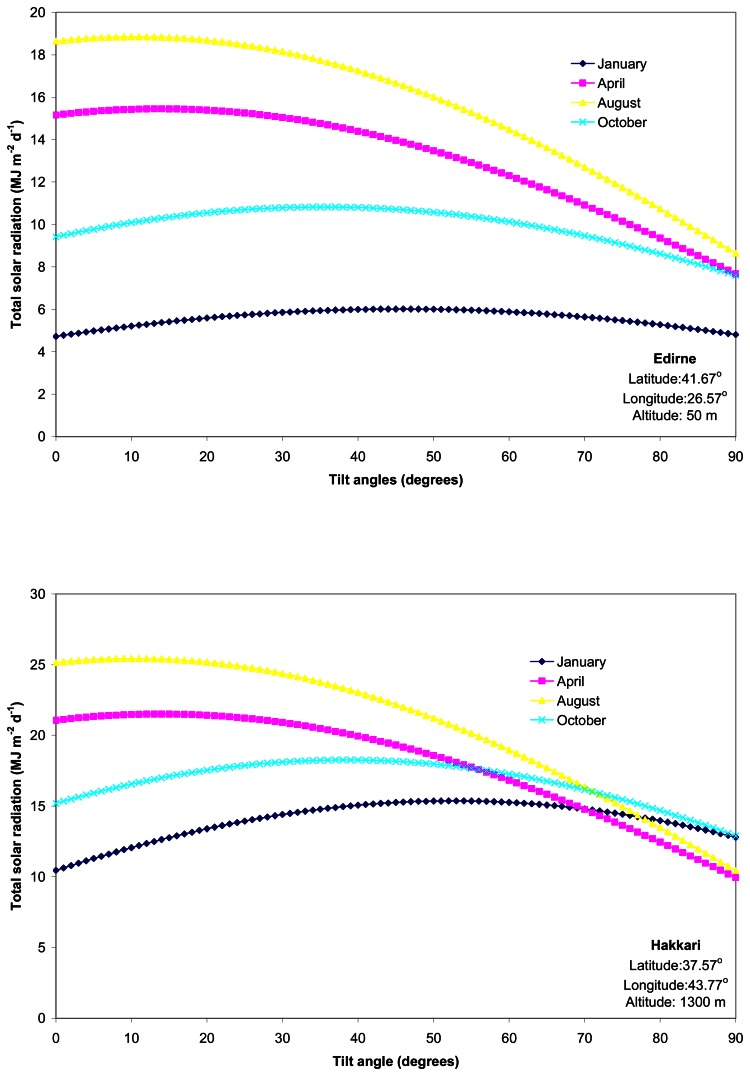

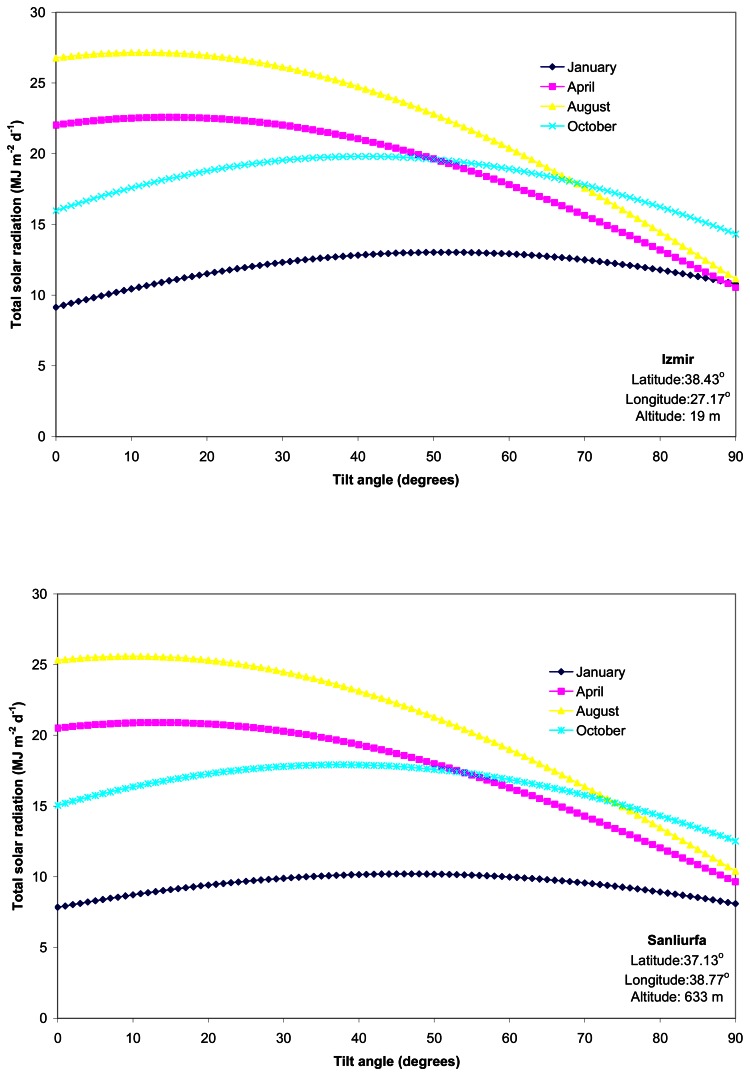

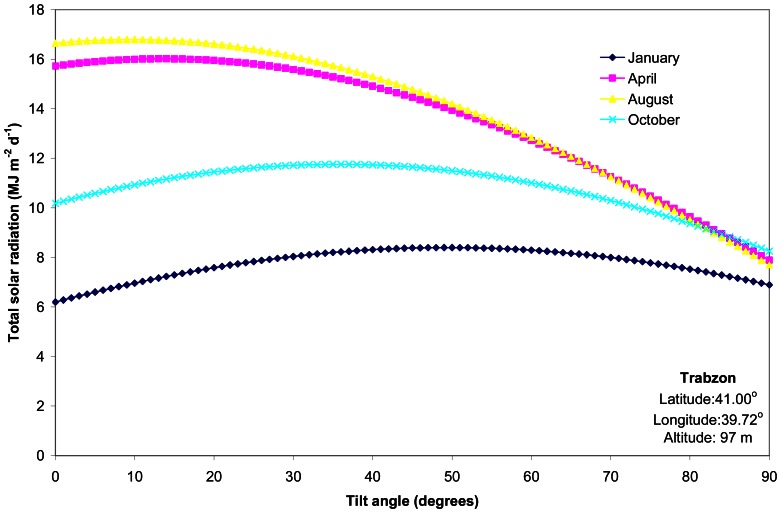
Seasonal changes in total solar radiation (*H*_T_, MJ m^-2^ d^-1^) on a south-facing solar collector according to tilt angles of 0 to 90° for seven cities selected as representatives of major climate zones in Turkey.

The amount of monthly average daily total solar radiation on a south facing collector along the tilt angle gradient of 0 to 90° was shown for seven locations selected as representatives of major climate zones of Turkey (Fig. 1). Total solar radiation varied from 5.19 MJ m^-2^ d^-1^ in December to 35.34 MJ m^-2^ d^-1^ in July, based on the observed dataset from the 158 locations across Turkey. The total solar radiation was high (*ca.* 29 MJ m^-2^ d^-1^) in Izmir and Antalya in August and decreased to *ca.* 4 MJ m^-2^ d^-1^ in Edirne in January. Our statistical exploration to model monthly optimum tilt angles of the south-facing collectors as a function of latitude, and day of the year over Turkey led to the following equation:

(14) $$\beta_{\text{opt}}=25.521438+26.838291\cos(-0.017844\lambda +1.013901n+7.527742)$$

**Figure 2.**
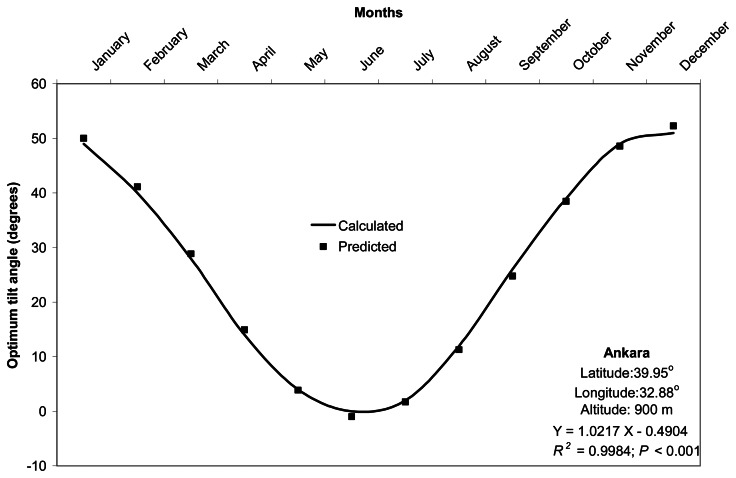

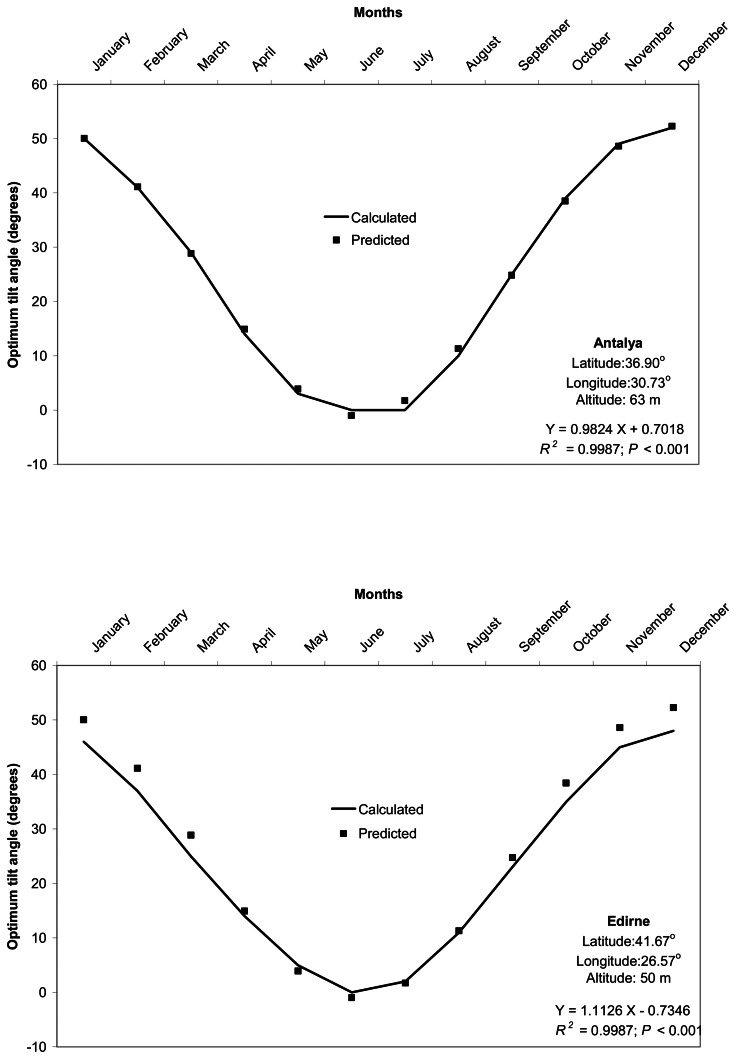

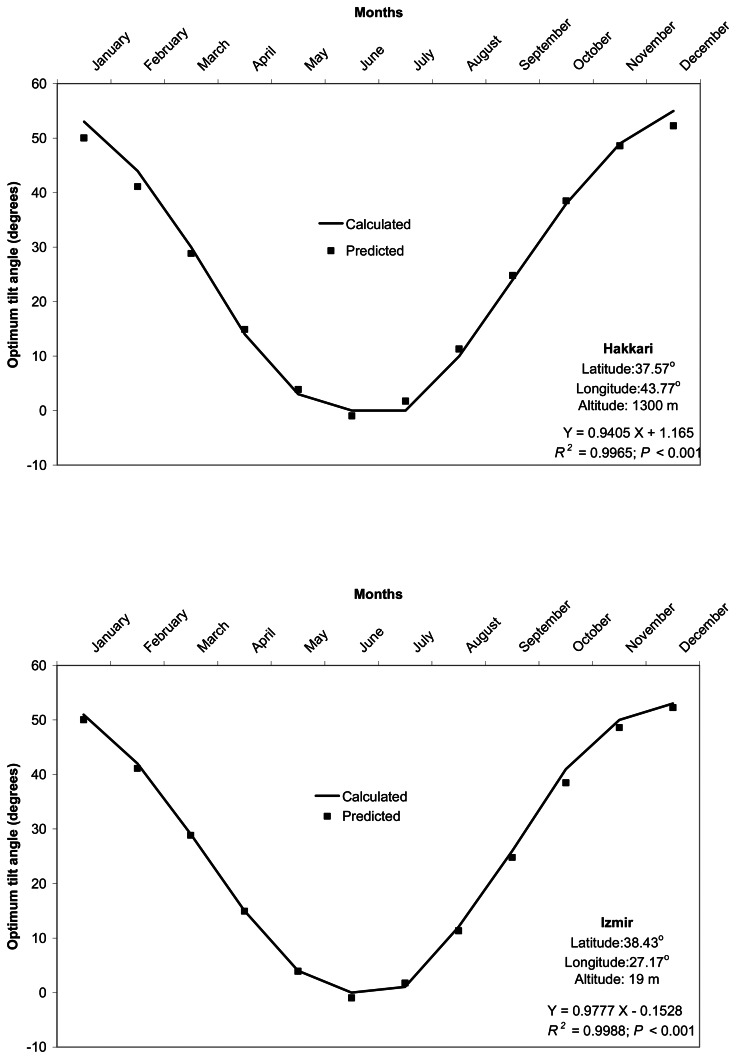

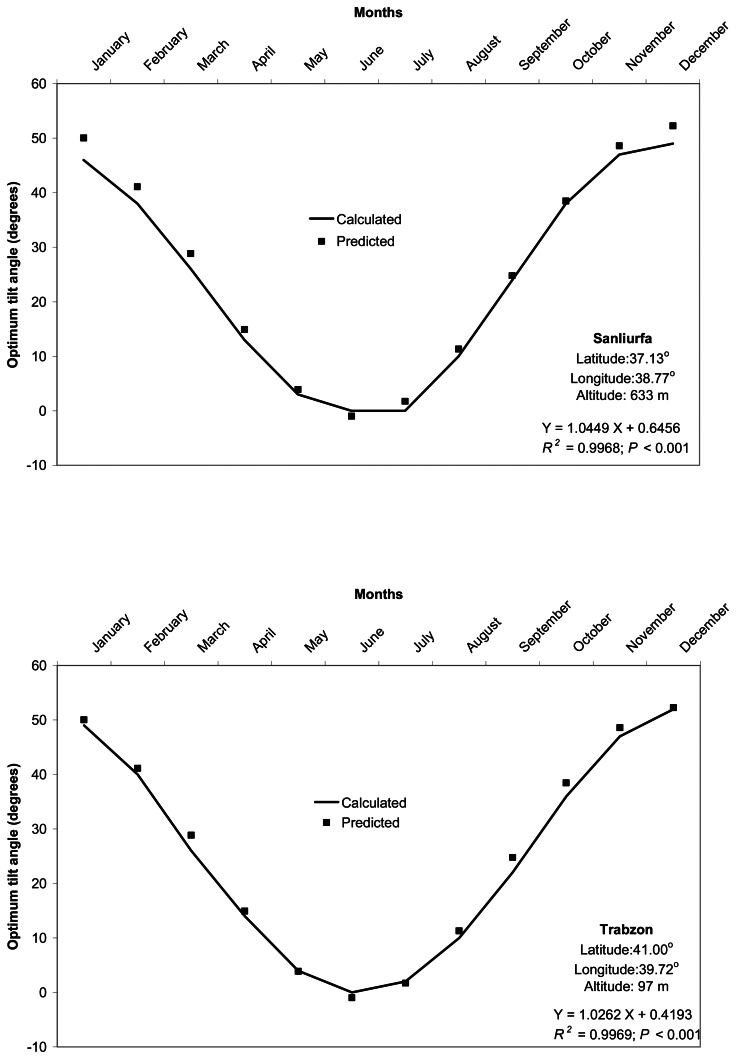
Monthly changes in calculated and predicted optimum tilt angles (degrees) for seven cities selected as representatives of major climate zones in Turkey.

Comparison of monthly optimum tilt angles calculated (by Equations 1 to 11) and predicted (by Equation 14) from the 158 locations resulted in a good agreement with *R*^2^ of 98.8%, and root mean square error (RMSE) of 2.06° (*P* < 0.001). Validation of monthly predicted versus calculated optimum tilt angles had minimum and maximum *R*^2^ values of 99.1% in Gumushane and 100% in Sinop, respectively (*P* < 0.001). Monthly optimum tilt angles predicted in this study deviated in a range of -8.98 to 8.02° from the calculated ones. Validation results were presented for seven cities selected as the representatives of major climate zones of Turkey in Fig. 2. It is noticeable that the observed optimum tilt angles for the months of June and July are equal to zero for some locations in Turkey.

### 3.2. Seasonal Optimum Tilt Angles

**Figure 3.**
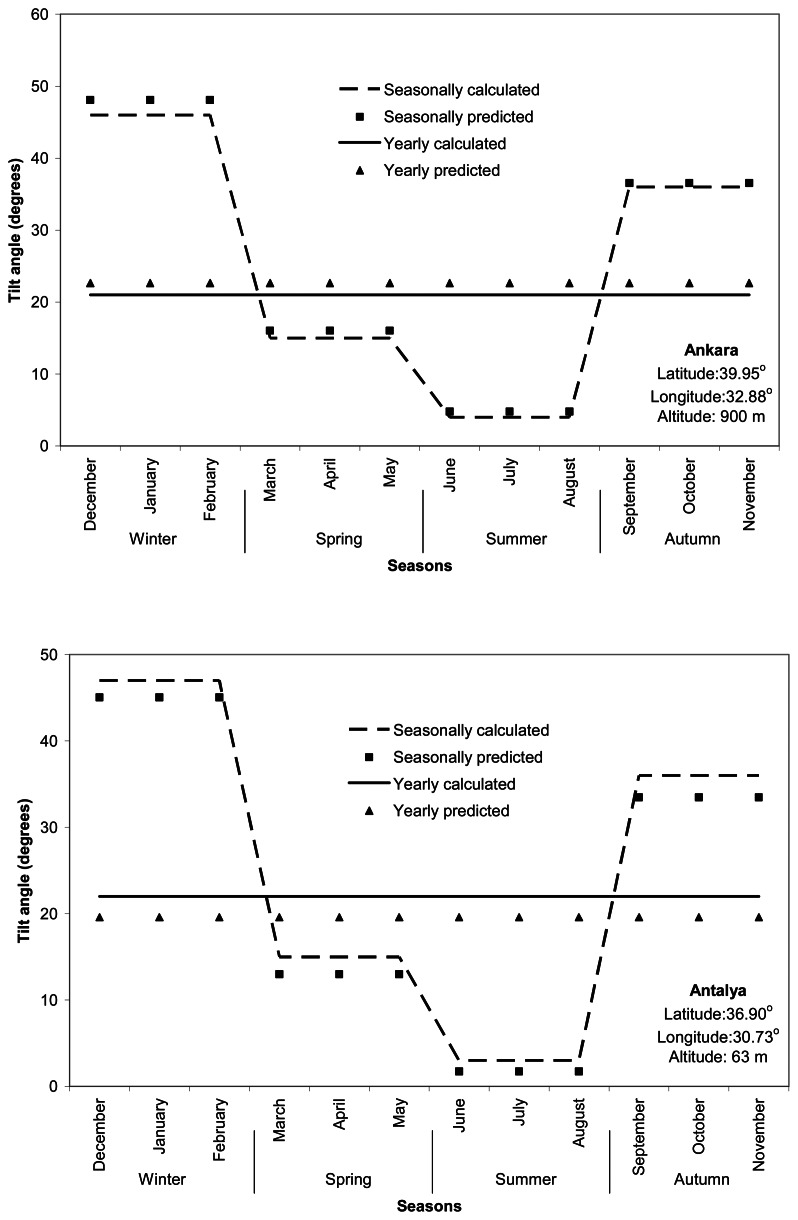

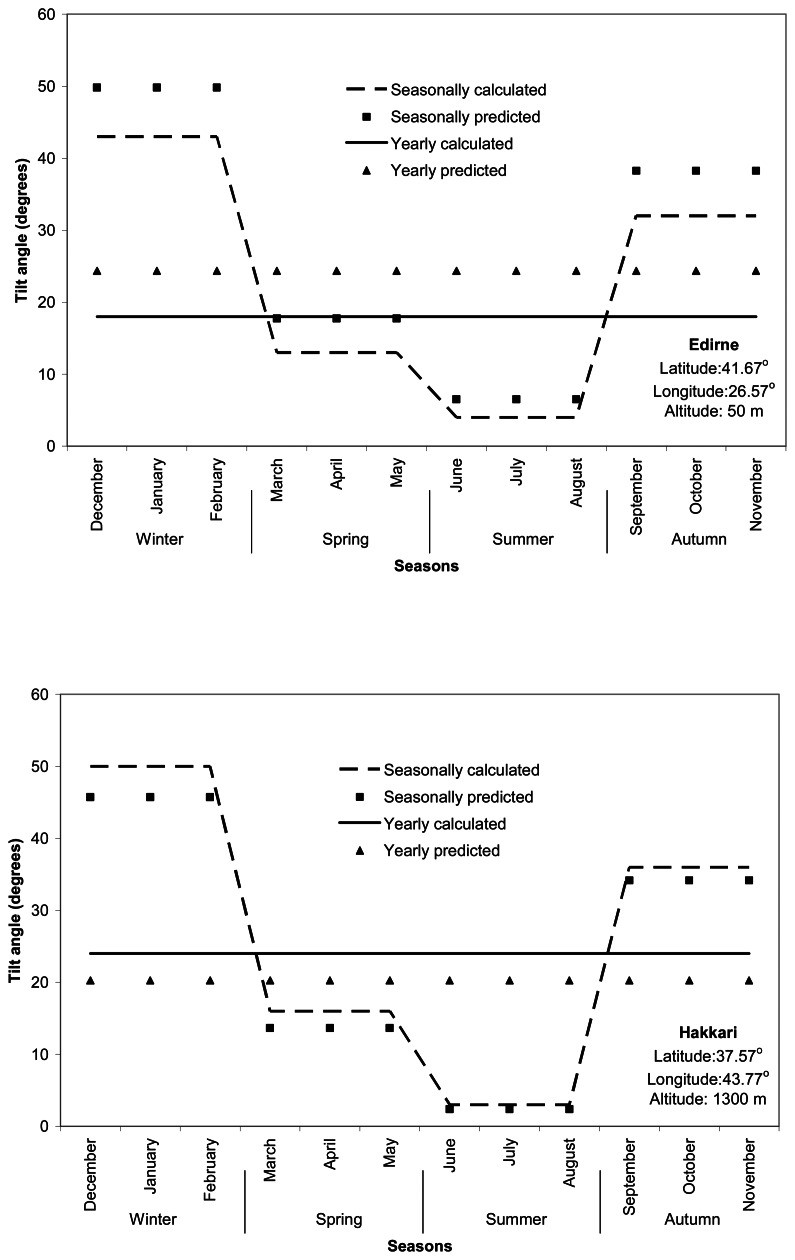

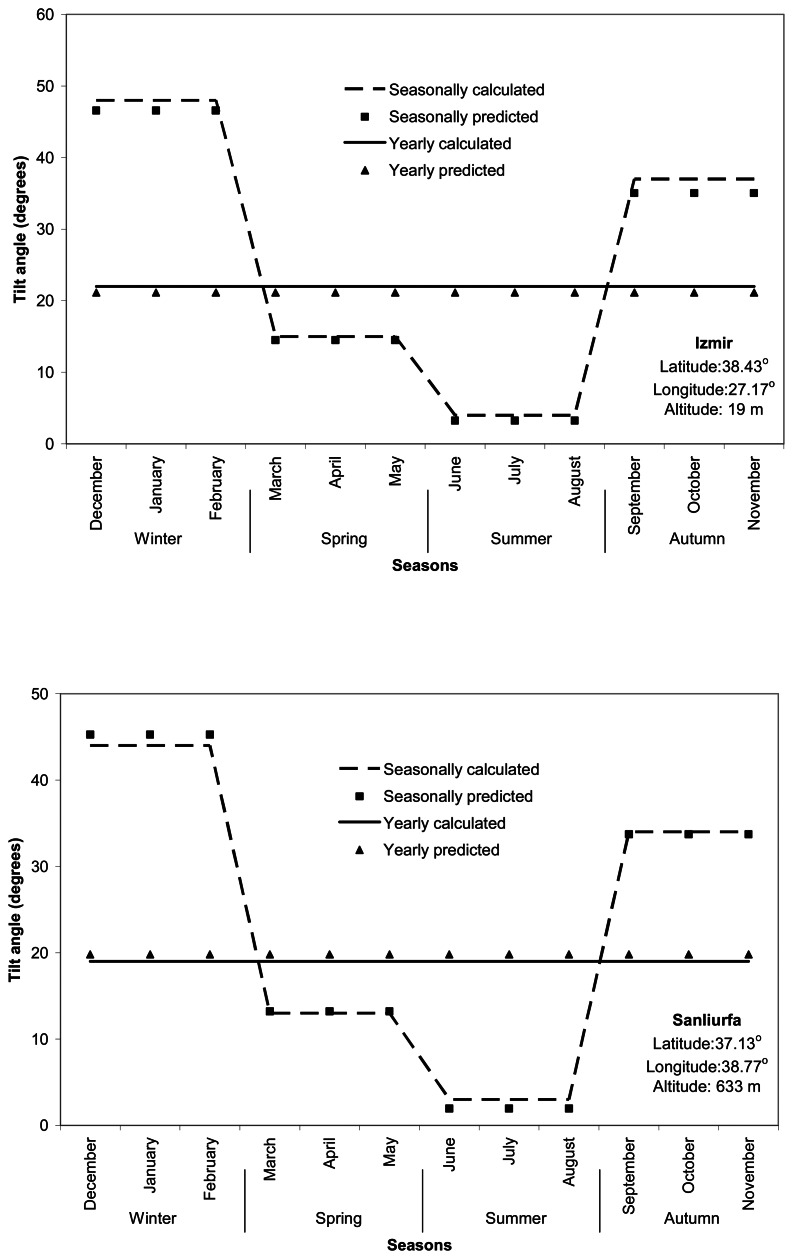

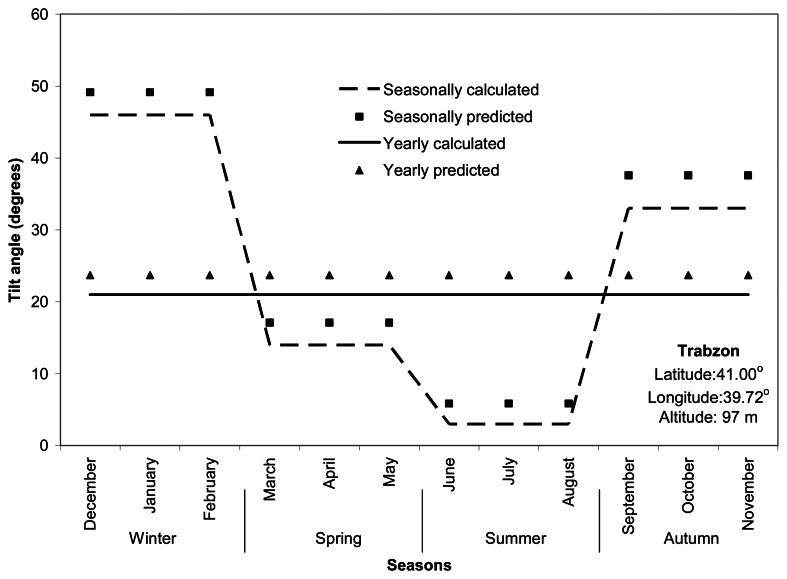
Seasonal and annual changes in calculated and predicted optimum tilt angles (degrees) for seven cities selected as representatives of major climate zones in Turkey.

The fact that adjusting the tilt angle to its monthly optimum values throughout the year does not seem to be practical gives rise to the consideration of changing the tilt angle once seasonally. The fixed optimum tilt angles for each season of winter (December to February), spring (March to May), summer (June to August), and autumn (September to November) were determined as an average of monthly solar radiation values for that season. The optimum tilt angles for the seasons were found to be *λ* -3.41° for autumn; *λ* + 8.14° for winter; *λ* - 23.92° for spring; and *λ* - 35.17° for summer in Turkey. The magnitude of the seasonal deviation between predicted and calculated tilt angles ranged from -9.81 to 7.21° in the winter; -4.87 to 4.75° in the spring; -1.37 to 2.83° in the summer; and -6.36 to 6.26° in the autumn, based on the 158 locations in Turkey. Seasonal and annual changes predicted and calculated for optimum tilt angles were given for the cities according to the seven major climate zones of Turkey in Fig. 3.

### 3.3. Annual Optimum Tilt Angles

The annual optimum tilt angles as a fixed value for the solar collectors varied from 16 to 29° over Turkey (Fig. 3). The annual optimum tilt angle may be used for the installation of stationary solar collector systems and can be based on the following relationship of λ - 17.31° throughout Turkey. The annual difference between predicted and calculated tilt angles was in the range of -8.26 to 6.36° for the 158 locations (Fig. 3).

### 3.4. Mapping Spatial Variability in Annual Optimum Tilt Angles

**Figure 4.**
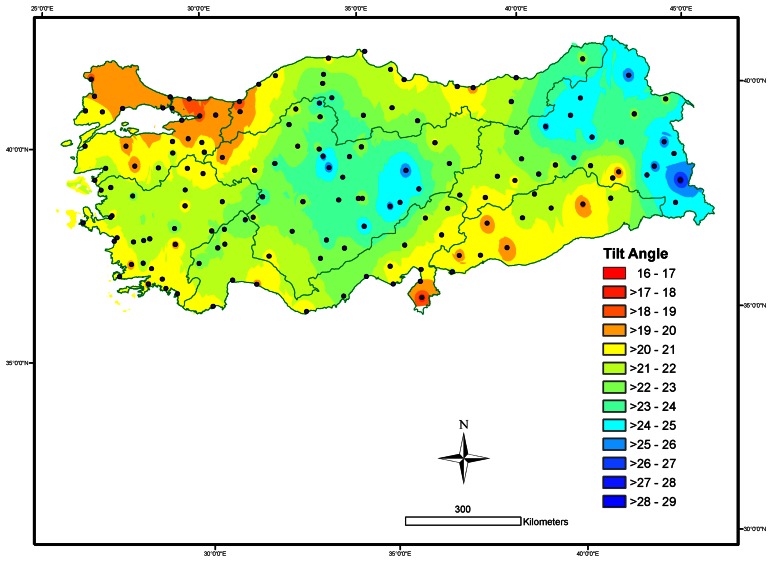
Surface map of mean annual optimum tilt angles (degrees) based on inverse distance weighting (IDW) interpolation with a grid resolution of 500 m x 500 m, and geographical distribution of 158 weather stations according to seven major climate zones of Turkey.

Spatial variation in the annual optimal tilt angles of the south-facing solar collectors was mapped based on the IDW interpolation technique over Turkey, with a grid resolution of 500 m x 500 m (Fig. 4). The IDW neighborhood was set to the 15 nearest neighbors with a minimum of ten neighbors for the data of annual optimum tilt angle, in order to capture small scale variability over Turkey. An optimized power value of 1.1168 was used in IDW to interpolate and visualize the predicted surface of annual optimum tilt angle, with the mean prediction error of 0.10, the root mean square prediction error of 1.85, and *R*^2^ of 34.5% for the spatial cross-validation (*P* < 0.001).

## 4. Conclusions

Monthly, seasonal and annual changes in optimum tilt angles for the solar collectors over Turkey were determined by using the geo-referenced datasets of monthly average daily global solar radiation from 158 cities and monthly diffuse radiation estimated by the empirical model by Tasdemiroglu and Sever [35]. Our results revealed that the optimum tilt angles exhibit a strong seasonal trend with respect to the amount of maximum daily insolation incident on the collector surface. Monthly average optimum tilt angles were reasonably well estimated as a sinusoidal function of latitude and the day of the year over Turkey. The optimum tilt angle was low in the summer and high in the autumn and winter. The maximum daily insolation is received on a south facing collector with tilt angles of (*λ* -8.14°) in the winter, whereas the maximum daily insolation is incident on a nearly horizontal surface (*λ* - 35.17°) in the summer. The spatially interpolated surfaces may guide the choice of annually optimal tilt angles for the fixed south-facing solar collectors, particularly where there is no information about solar radiation across Turkey.
